# Rethinking Power Efficiency for Next-Generation Processor-Free Sensing Devices

**DOI:** 10.3390/s22083074

**Published:** 2022-04-16

**Authors:** Yihang Song, Songfan Li, Chong Zhang, Shengyu Li, Li Lu

**Affiliations:** School of Computer Science and Engineering, University of Electronic Science and Technology of China, Qingshuihe Campus, Chengdu 611731, China; songyihang@std.uestc.edu.cn (Y.S.); songfanli@std.uestc.edu.cn (S.L.); zhangchong@std.uestc.edu.cn (C.Z.); lishengyu@std.uestc.edu.cn (S.L.)

**Keywords:** backscatter sensing devices, power optimization, I^2^C, processor-free devices

## Abstract

The last decade has seen significant advances in power optimization for IoT sensors. The conventional wisdom considers that if we reduce the power consumption of each component (e.g., processor, radio) into μW-level of power, the IoT sensors could achieve overall ultra-low power consumption. However, we show that this conventional wisdom is overturned, as bus communication can take significant power for exchanging data between each component. In this paper, we analyze the power efficiency of bus communication and ask whether it is possible to reduce the power consumption for bus communication. We observe that existing bus architectures in mainstream IoT devices can be classified into either push-pull or open-drain architecture. push-pull only adapts to unidirectional communication, whereas open-drain inherently fits for bidirectional communication which benefits simplifying bus topology and reducing hardware costs. However, open-drain consumes more power than push-pull due to the high leakage current consumption while communicating on the bus. We present *Turbo*, a novel approach introducing low power to the open-drain based buses by reducing the leakage current created on the bus. We instantiate *Turbo* on I${}^{2}$C bus and evaluate it with commercial off-the-shelf (COTS) sensors. The results show a 76.9% improvement in power efficiency in I${}^{2}$C communication.

## 1. Introduction

The last decade has seen significant advances in power optimization for IoT (Internet of Things) sensing devices. Specifically, the idea of passive communication eliminates the need for power-starving carrier generation in wireless communication, and conveys information by backscattering ambient signals, thereby reducing the power consumption of radio components down to microwatts (μW). Based on passive communication, energy budgets for transmitting data to the gateway for further processing, become cheaper than locally processing it at the sensing device This fact fascinates a number of interesting researches to offload the processor, which is conventionally used to perform functions including sensor control, data processing and communication control at sensing devices, to the IoT gateway [1,2,3], giving rise to processor-free IoT sensing devices. This trend draws a compelling vision for us—given that various sensor ICs are triggered infrequently, and thus often operate at the μW-level of power [4] or even fully passive [5], the next-generation backscatter sensing devices are expected to have overall ultra-low power consumption and are able to be designed as passive and ubiquitous because all of the components are with μW-level of power. Besides, the processor-free device achieves a distinctive anonymity sensing method, which can prevent the unauthorized third party from revealing the identities of the communication parties [6], as there is no information related to the identity of the user, such as programs, wireless connection settings, and sensing logs.

**Figure 1:**
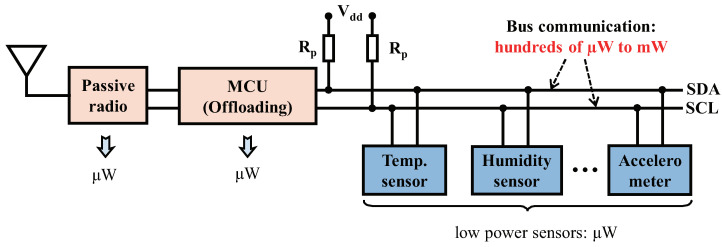
Although backscatter and offloading techniques could let components such as radio and the processor achieve ultra-low power, the bus communication to convey data among those components consumes significant power, which cannot be ignored in such backscatter devices.

Nevertheless, improving the power efficiency of such processor-free sensing devices is still challenging, because bus communication can take significant power for exchanging data between each component. The bus communication represents a data transmission process executed on the digital bus (e.g., the I${}^{2}$C bus and the SPI bus) of the sensing devices. In traditional embedded systems, it is reasonable to suppose that bus communication consumes insignificant power since radios and processors account for the majority of power consumption, which drains at the level of milliwatts (mW). However, we observe that the power consumption of bus communication is increasingly becoming a predictable bottleneck for such processor-free sensing devices. For example, the advanced backscatter radio designs show power consumptions of about 15μW [7,8], whereas an I${}^{2}$C bus can eat power of 214μW when operates at the standard mode (Section 2.1 in detail), 14.2× of the power consumption of backscatter radios, as shown in Figure 1. Similarly, SMbus also consumes hundreds of μWs of power, as it requires similar pull-up resistors and supply voltages as I${}^{2}$C [9,10]. The power-consuming bus communication between the major components makes it challenging to further optimize the power consumption of processor-free sensing devices. In addition, the high power consumption of bus communication also constraints the number of sensors equipped on IoT end devices.

In this paper, we rethink the power efficiency for future processor-free IoT sensing devices and ask whether it is possible to reduce the power consumption for bus communication. We observe that existing bus architectures in mainstream IoT devices can be classified into two categories (Section 2.1 in detail). (1) push-pull: although the push-pull architecture features relatively lower power consumption than the other, it only adapts to unidirectional communication, often complicating the bus topology and limiting the scalablity of IoT sensing devices. (2) open-drain: the open-drain architecture inherently fits for bidirectional bus communication, thus having more flexibility in connection with sensors. For example, I${}^{2}$C is an open-drain-based bus that can easily connect a series of sensors via only two lines, which benefits simplifying topology in architecture and reducing hardware cost. However, the open-drain architecture has a significant leakage current consumption which leads to hundreds of μWs of power. As a conclusion, while there is no perfect bus architecture yet, if the power consumption problem of open-drain can be resolved, this architecture could become the most suitable choice for backscatter devices.

We advocate *Turbo*, a novel approach introducing low power to the open-drain based buses (e.g., I${}^{2}$C, SMbus) by reducing the leakage current created on pull-up resistors $R_{p}$ during the bus communication. We instantiate *Turbo* on I${}^{2}$C bus to achieve a concrete illustration. To limit the current, the basic way relies on increasing $R_{p}$ resistance, thereby limiting the current. The higher resistance, however, incurs worse signal distortion and may corrupt the communication. The I${}^{2}$C standard states a range of the resistance to limit the distortion, and thus restricts the maximum resistance. We show that the leakage consumption in many cases is significant yet, in spite of the selection of maximum resistance.

To break above barrier, theoretically, we can leverage a signal waveshaping module that reforms the waveform of distorted signals to be regular. In this way, a higher resistance that breaks the constraint of the maximum resistance value can be chosen to further lower the leakage consumption. The consequent signal distortion resulted from the high resistance is corrected by the waveshaping module before to be received by the target sensor IC. However, the waveshaping module introduces an extra challenge and prevents the I${}^{2}$C communication. The bus lines are specified to be bidirectional that transmits and receives signals on the same line. Oppositely, the desired waveshaper is an unidirectional function block and is only able to receive signals. Therefore, the waveshaper has to be placed on the Rx branch that is at inside of the chip. To implement the waveshaper, the design of the bus interfaces inside chips has to be modified for adding the waveshaper, which results in incompatible with existing chips.

We handle the challenge by our observation that the Rx data register in existing bus interfaces has a concealed function, in which the basic storage elements can be treated as the waveshaping module. Thus, the observation exempts us from changing the designs of existing sensors and allows us to achieve *Turbo* with the off-the-shelf sensors. The only change is the need of increasing the pull-up resistance on the I${}^{2}$C bus. While *Turbo* brings the dawn of low power I${}^{2}$C communication, two key questions still remain.

- First, the signal distortion becomes worse along with the increasing of resistance. Is the communication still robust with *Turbo*? At a certain resistor, the signal may not be effectively recovered by the waveshaping module so that I${}^{2}$C communication fails. How to decide a resistance selection range in *Turbo*?
- Second, while the power consumption decreases along with the increasing of resistance, we observe that the communication data rate is reduced as well. Why does the data rate decrease? What is the relationship between the power consumption and the data rate and how to formulate it? How to select an adequate resistor value to make a trade-off between the power consumption and the data rate?

We propose three technical solutions to address the above challenges.

- First, we determine the effective resistance selection range (Section 3.1) by investigations to analyze the signal distortions over different resistances.
- Second, we build a physical signal model to describe the relationship between the power consumption (Section 3.3) and the data rate (Section 3.2). The model is based on a heuristic method and consider physical signal features. We also find the reason of the data rate reduction, i.e., the higher resistance incurs the increase of required time for each bit data transmission.
- Third, we formulate an optimal resistance problem (Section 3.4) to seek the best solution to reduce the power consumption as much as possible, meanwhile sacrificing the data rate as less as possible. At the optimal resistance, the bit energy budget is minimized in I${}^{2}$C communication.

We conduct experiments to verify the feasibility of *Turbo* with off-the-shelf sensors. Then, we evaluate *Turbo* by both single-sensor and multi-sensors experiments, in which 1.6 megabytes of data are transmitted to each of the four sensors. The results show that *Turbo* inherits robust communication from the standard. *Turbo* achieves 0% bit error rate (BER) within the effective resistance selection range. Moreover, when the optimal resistance is selected, 76.9% of the bit energy budget is reduced on average while sacrificing 64.5% of the data rate. This demonstrate that although the bit rate is decreased, the total energy consumption to perform I${}^{2}$C communication is reduced.

The contributions of *Turbo* are in three folds.

- We first point out the problem of high power consumption of open-drain bus communication, which is widely used in low-power sensing devices, and we propose the basic idea of reducing leakage current of the open-drain architecture to reduce the energy overhead.
- We propose technical solutions to the challenges hindering the realization of our basic idea, including signal distortion, and the reduction of data rate.
- We verify the feasibility and evaluate the performance of *Turbo* with both hardware prototype and simulation.

## 2. Preliminary

### 2.1. Background of Bus Architecture

To better understand *Turbo*, first we introduce two types of output architectures that are commonly used in bus communication, then we illustrate the leakage current in *open drain*-based buses.

**Figure 2:**
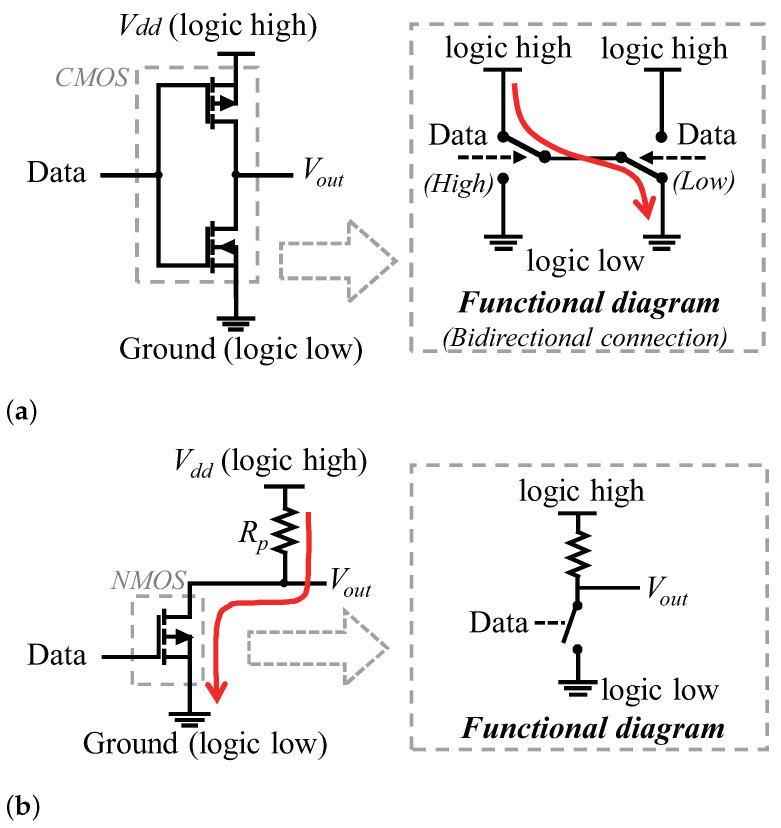
*Push–pull* and open-drain architectures. (**a**) The push-pull architecture. The CMOS act as a single-pole double-throw switch to connect the $V_{out}$ to either $V_{dd}$ or ground. When two push–pull outputs are connected, it is possible to cause a short circuit if one of the outputs is high and the other is low. (**b**) The open-drain architecture. The NMOS transistor acts as a switch. The leakage current (red solid arrow) occurs when the switch is closed and $V_{out}$ is logic low.

The *push–pull* architecture leverages a typical CMOS configuration, as is shown in Figure 2a. Each CMOS acts as a single-pole double-throw (SPDT) switch, which is driven by the transmitting data. When the logic level of data is high, the NMOS is turned on and connects the $V_{out}$ to $V_{dd}$ (logic high). When the logic level of data is low, the PMOS is turned on, and then the $V_{out}$ is connected to ground and becomes logic low. However, two push–pull outputs cannot be connected together, since the current will flow freely from $V_{dd}$ to ground if one output is high and the other is low. Thus, the *push–pull* architecture cannot achieve bidirectional communication.

The open-drain architecture consists of an NMOS transistor and a pull-up resistor $R_{p}$, as shown in Figure 2b. In open-drain, the transistor is driven by the data and works like a single-pole single-throw (SPST) switch. If the switch is open (disconnected), the output $V_{out}$ gets pulled up to $V_{dd}$ through the resistor $R_{p}$ and begins to be logic high. If the switch is closed, the output is connected to the ground and becomes logic low, and due to the existence of the pull-up resistor, the open-drain structure will not cause a short circuit when used for bidirectional communication.

**Figure 3:**
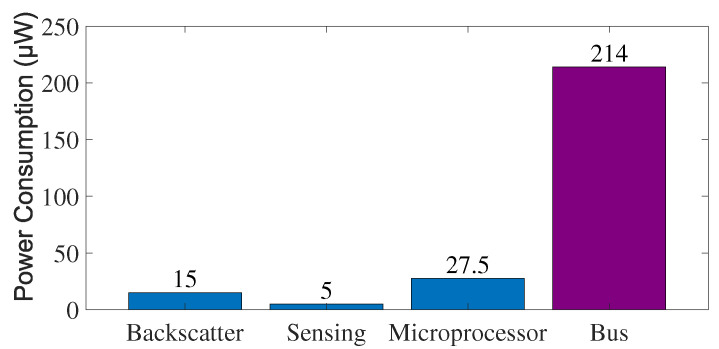
The power consumption comparison of typical components and functions on backscatter sensors, including the backscatter communication, sensors, processor functions (simplified using the R2B technique) and bus communication.

However, the open-drain-based buses suffer from a leakage current problem, which increases their power budget of data transmission. Let us take I${}^{2}$C bus as an example to illustrate. I${}^{2}$C bus exploits the open-drain architecture in both SDA and SCL bus lines to realize bidirectional communication regardless of the master or slave roles. Nevertheless, the open-drain output brings the concern of notable leakage current when the output is logic low, as there is current which flows through the resistor $R_{p}$, and then the switch to ground. For example, a typical configuration ($R_{p}=3.3\;k\Omega$ and $V_{dd}=3.3\;V$) of I${}^{2}$C used on conventional embedded systems leads to a power consumption of more than 3 mW; further, on recent backscatter sensing devices such as WISP [11], the power consumption of I${}^{2}$C bus communication can be reduced to hundreds of μW. However, this is far from realizing μW-level overall power consumption on backscatter sensing devices. According to our experiment, the I${}^{2}$C bus communication on WISP ($R_{p}=10\;k\Omega$ and $V_{dd}=1.8$ V) consumes 51.41 μJ to transmit 2 KB data with this configuration, yielding a power consumption of 214μW, which are 14.2×, 42× and 7.8× of the power consumption of typical backscatter communication techniques [7,8], sensors [4] and simplified local processor functions [3], as shown in Figure 3.

### 2.2. Challenges of Turbo

**Challenge 1—Signal Distortion.** To reduce the leakage current, the basic way is to increase the pull-up resistor $R_{p}$ or lower the positive voltage $V_{dd}$. As this voltage is directly specified by the sensors connected to the bus and is typically fixed in practical use, the general purpose method is to increase $R_{p}$. Higher $R_{p}$, however, incurs worse distortion on signals. This is because, in practice, the bus lines and the adjacent circuit unavoidably construct an equivalent parasitic capacitor. The capacitor $C_{b}$ and the resistor $R_{p}$ constitute an RC circuit that makes the rise edge of square wave signals slow, and thus deforms the waveform of I${}^{2}$C voltage signals. To prevent the distortion, the I${}^{2}$C standard limits the maximum signal rising time $t_{r}\leq$ 1 μs, which in other words, represents the maximum standard resistance $R_{p(max\_std)}$. Empirically, the value of $R_{p(max\_std)}$ is around 10 KΩ that often results in hundreds of μA leakage current and is still a huge overhead to the backscatter devices.

**Figure 4:**
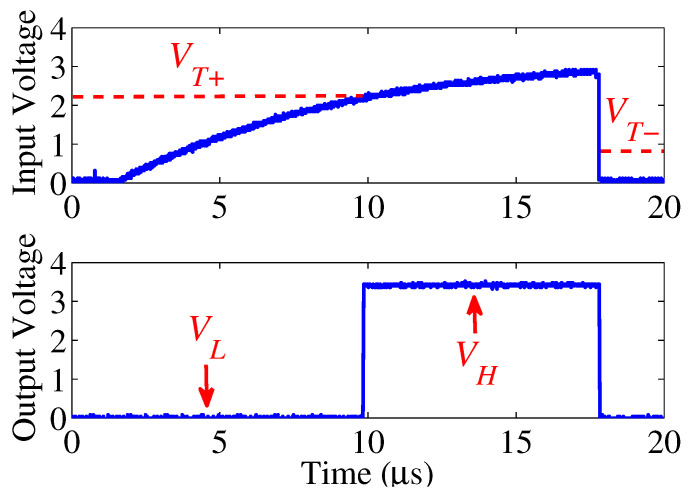
The input voltage is the distorted bus signal and the output is the standard I${}^{2}$C signal that the signal rising time is obviously less than 1 μs.

**Solution.** Our insight is that the distorted signals can be recovered by a waveshaping module that converts the slow rise edge to a fast edge, so that the signal waveform meets the time requirement. In this way, the selected $R_{p}$ can exceed the maximum standard resistance $R_{p(max\_std)}$ to further reduce the leakage current while the on-bus sensors can receive standard signal which is being recovered by the waveshaper. The waveshaping module can be implemented by a Schmitt trigger [12]. As shown in Figure 4, the Schmitt trigger contains two thresholds, $V_{T+}$ and $V_{T-}$, respectively. When the input is higher than the upper threshold $V_{T+}$, the output of the trigger is $V_{H}$ = $V_{dd}$. Oppositely, when the input is below the lower threshold $V_{T-}$, the output is $V_{L}$ = 0 V. The dual threshold feature also improves the noise immunity from a single threshold trigger, so that the distorted waveform can be converted to the closely ideal waveform.

**Figure 5:**
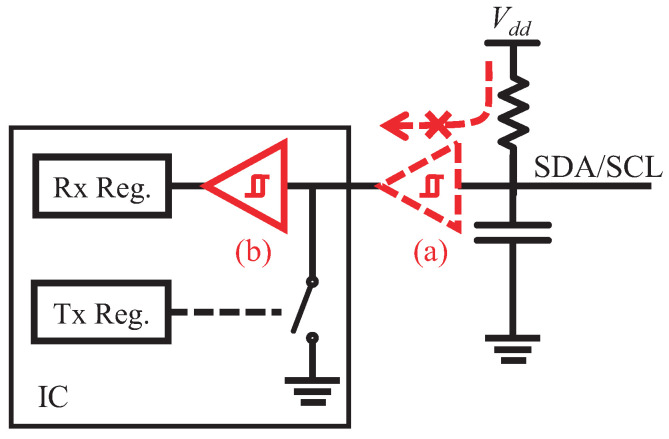
We expect that the waveshaping module is put at the place (a) outside the chip, but the module will block the current so that the chip cannot transmit data to the bus. The placement (b) on the receive branch is adequate, but it requires modifying the chip design.

**Challenge 2—Placement Problem.** The waveshaping module, however, incurs a consequent problem that it will block the signal transmission on the bus. The I${}^{2}$C bus lines are bi-directional, where all chips receive and transmit signals via single bus line. The placement of the Schmitt trigger on the bus line will divide the line, so that the current from the source cannot pass through the trigger and the Tx circuit is ineffective, as described in Figure 5. The reason stems from the open-drain architecture, in which the output source is the voltage source $V_{dd}$ at the pull-up resistor side, while the transmit (Tx) circuit actually controls whether collecting the voltage or releasing it (imagine that you are operating a water gate at the bottom of an impounding reservoir). Unfortunately, there are no designs for bi-directional triggers that allow current through and can be used in I${}^{2}$C communication to the best of our knowledge. Accordingly, the trigger has to be placed on the receive (Rx) branch. To do this, however, we have to modify the circuit inside the integrated circuit (IC), which incurs *Turbo* incompatible with existing ICs.

**Figure 6:**
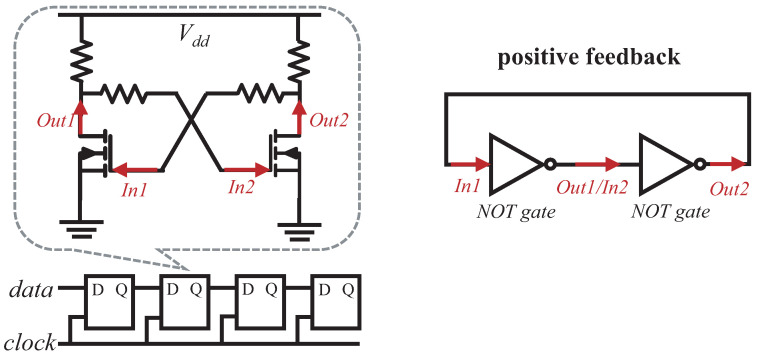
Rx register circuit diagram (4 bit). The kernel of a flip-flop (in the dashed box) is the same as a Schmitt trigger.

**Solution.** We tackle the placement problem according to an observation that existing ICs contain a potential waveshaping function. The arriving signals are directly received by the Rx register inside the ICs. We observe that the register is provided with the function of a Schmitt trigger and has the ability to recognize correct data from distorted signals. While it looks like magic, we observe that the reason is scientific. The register consists of several flip-flops that are used as data storage elements, in which each flip-flop stores a single bit of data [13], as shown in Figure 6. The kernel of a flip-flop is same to the trigger since they are both the bistable multivibrator [14]. Essentially, the multivibrator contains two NMOS transistors, in which the output of each transistor becomes the input of the other transistor. Each transistor acts as a NOT gate and the output of one transistor finally comes back as its input, which is a typical positive feedback scheme and is the fundamental idea of Schmitt triggers.

Although flip-flops have various types and different implementations [15], the kernel architectures are similar that contain the positive feedback, and hence have the functionality of waveshaping. However, the waveshaping performance of registers would be poorer than the Schmitt trigger because the circuit is optimized for data storage rather than the waveshaping. Fortunately, we observe that many existing ICs have already adopted Schmitt triggers at the input [16,17,18] for anti-noise applications. Note that their goal of using the trigger is different from *Turbo*. These ICs employ the trigger to improve the noise immunity that supports higher data rate in communication. Conversely, *Turbo* leverages the Schmitt trigger to correct the distorted signals in order to reduce power consumption that results in a lower data rate. In Section 4.3, we evaluate *Turbo* for both types of ICs (i.e., with or without Schmitt triggers).

## 3. Optimizing the Leakage Current

In this portion, we target the investigation of an effective resistance selection range and build a physical signal model to illustrate the relationship between the power consumption and the data rate over the pull-up resistance $R_{p}$.

**Figure 7:**
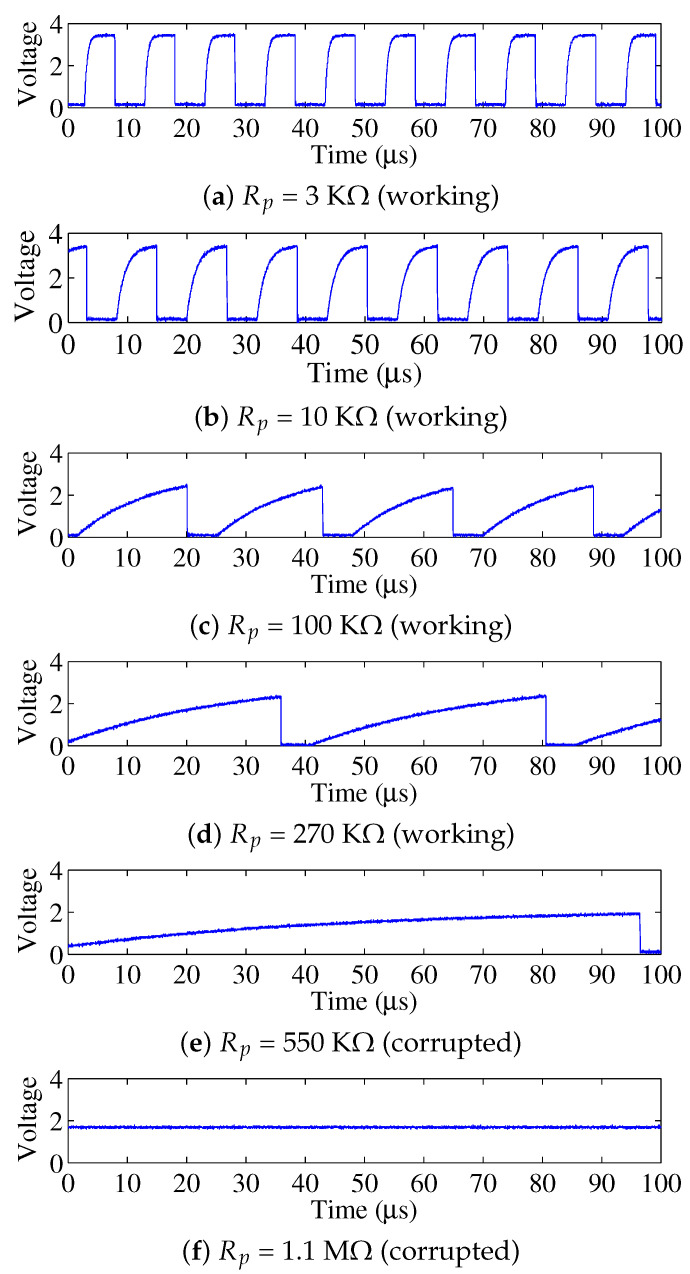
SCL waveform (before the waveshaper) over different pull-up resistances. I${}^{2}$C communication works in case of (**a**–**d**), while the communication is corrupted in case (**e**,**f**).

To better understand the model, we use a heuristic way to illustrate the modeling. We conduct an experiment to observe the I${}^{2}$C communication at different $R_{p}$. We consider the experiment on an I${}^{2}$C bus with $C_{b}$ = 142 pF and $V_{dd}$ = 3.3 V. A processor which acts as the master sends pre-defined data via the bus to another processor which acts as the slave. Once the data has been sent, the slave checks the data correctness and lights a light-emitting diode (LED) for indicating whether the data transmissions are successfully completed. We repeat the experiment over different $R_{p}$ and show the observed SCL waveform in Figure 7. We do not show SDA because the open-drain architectures of SDA and SCL are the same, but the SCL is more critical in that it defines whether I${}^{2}$C communication can be performed, while the SDA waveform only represents the transmitted data. The results introduce two key observations to us.

### 3.1. Resistance Selection Range

**Figure 8:**
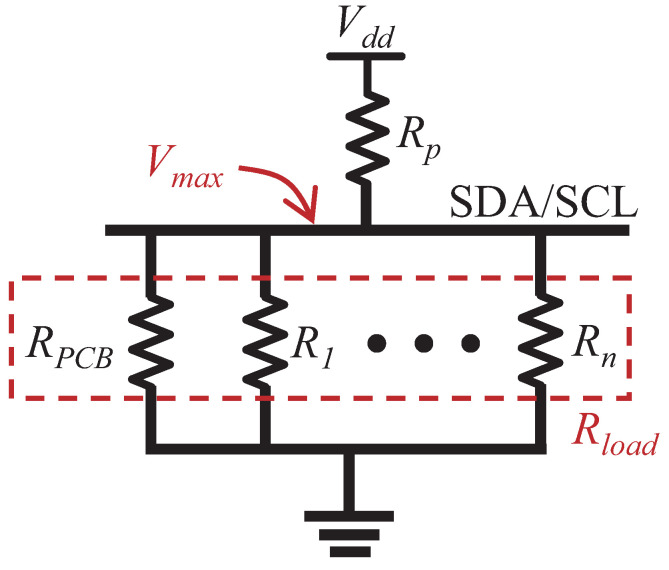
Equivalent circuit of the SDA/SCL line. $R_{i}$ refers to the equivalent resistance of the *i*th IC (*i* = 1 to *n*) when the open-drain states the high impedance. $R_{PCB}$ is related to the materials of making the PCB.

A selection range of $R_{p}$ exists in *Turbo*. We observe that the maximum peak voltage $V_{max}$ decreases along with the increase of $R_{p}$. For example, $V_{max}$ is 3.3 V, 2.52 V and 2.36 V when the $R_{p}$ is 10 kΩ, 100 kΩ and 270 kΩ, respectively, in Figure 7. The communication is corrupted when $V_{max}$ is less than the logic high threshold $V_{high}$. For example, the $V_{max}$ becomes 2 V and 1.72 V when the $R_{p}$ = 550 kΩ and 1.1 MΩ, respectively, while $V_{high}$ = 2.31 V. The reduction of $V_{max}$ stems from the reason that the connected sensor ICs on the bus are equivalent to a load resistance $R_{load}$, as shown in Figure 8. Increasing $R_{p}$ results in smaller $V_{max}$ since higher $R_{p}$ divides more voltages. Thus, the value of $R_{p}$ is restricted by the condition of $V_{max}>V_{high}=0.7\;V_{dd}$. With this restriction known, we can get, Equation (1).

(1) $$R_{p(max\_std)}<R_{p}<\frac{3}{7}R_{load}=\frac{3R_{PCB}}{7(1+R_{PCB}\;\cdot \;\sum_{i=1}^{n}G_{i})}$$

where $R_{p(max\_std)}$ is the maximum resistance recommended by the I${}^{2}$C standard, $G_{i}=\frac{1}{R_{i}}$ refers to the equivalent conductance of the sensor IC *i*, $R_{PCB}$ is the resistance of PCB, and *n* is the number of sensor ICs on the bus. According to Equation (1), we also observe that the maximum $R_{p}$ decreases along with the increase of the number of connected chips.

### 3.2. Data Rate Reduction

We observe that the communication data rate decreases as the duration to transmit each bit increases. For example, the bit rate is 100 Kbit/s, 84.7 Kbit/s, 43.7 Kbit/s and 22.4 Kbit/s, respectively, in Figure 7a–d. This happens because the PCB has equivalent capacitance $C_{b}$, and a higher $R_{p}$ increases the RC value of bus lines and thus the voltage rises slowly. When the output is above the logic high threshold $V_{high}$ and this clock period is passed, the output will be tripped to the logic low voltage after hitting $V_{high}$. Thus, the time to transmit one bit is formulated as Equation (2).

(2) $$t_{bit}=\underset{t_{low}}{\underset{︸}{\frac{1}{2f_{SCL}}}}+\underset{t_{charge}}{\underset{︸}{R_{p}\;\cdot \;C_{b}\;\cdot \;ln\left(\frac{V_{max}}{V_{max}-V_{high}}\right)}}+\Delta t$$

where $f_{SCL}$ is the clock frequency on the SCL line, $R_{p}\;\cdot \;C_{b}\;\cdot \;ln\left(\frac{V_{max}}{V_{max}-V_{high}}\right)$ is the time required for charging the RC circuit, which is composed of the equivalent capacitor of PCB and the pull-up resistor, Δt refers to the duration between the voltage reaching $V_{high}$ and then becoming low. As we observe that Δt is typically very small compared to the clock period, for convenience, Δt can be negligible. Therefore, the maximum bit (data) rate is calculated as:

(3) $$\begin{array}{ccc} B_{rate} & \approx & \frac{2f_{SCL}}{1+2f_{SCL}\;\cdot \;R_{p}\;\cdot \;C_{b}\;\cdot \;ln\left(\frac{V_{max}}{V_{max}-V_{high}}\right)}\\ & = & \frac{2f_{SCL}}{1-2f_{SCL}\;\cdot \;R_{p}\;\cdot \;C_{b}\;\cdot \;ln\left(0.3-0.7\;\cdot \;\frac{R_{p}}{R_{load}}\right)} \end{array}$$

### 3.3. Building Signal Model

Based on the two observations above, we build a physical signal model to explore the relationship between the power consumption and the bit rate. We first formulate the energy budget for single bit transmissions and then derive the relationship to the bit rate.

**Figure 9:**
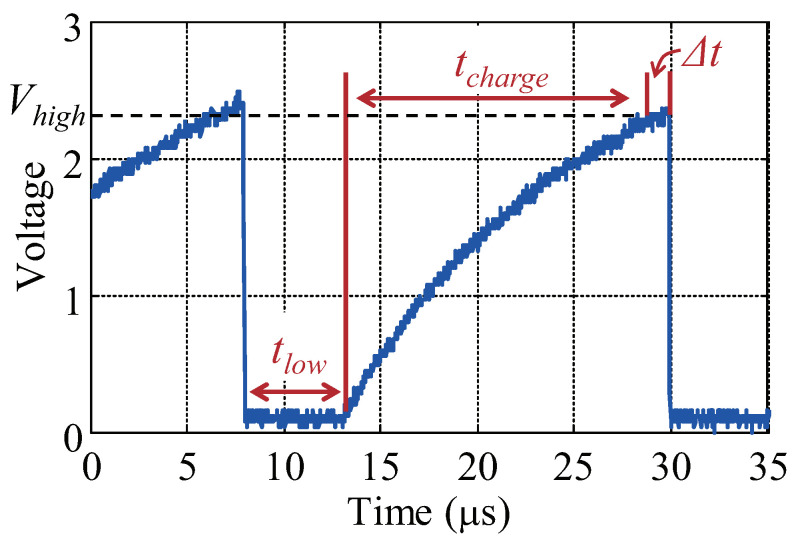
The time for each bit transmission.

The single bit energy budget consists of the energy consumption on the two lines (SCL and SDA). First, the consumption on the SCL line comprises two portions. One is the leakage current consumption (the $t_{low}$ part in Figure 9), while the other is the switching dissipation (the $t_{charge}$ part). The switching dissipation $E_{spbit}$ refers to the energy stored in the bus capacitor $C_{b}$ and further released to the ground. Second, the bit energy budget on the SDA line is related to the bit data. If the data is “bit-0”, the SDA line stays logic low during the bit period and the leakage current stays for a period of $t_{bit}$. If the data is “bit-1”, the line is charged to logic high and the switching power dissipation of $E_{spbit}$ happens. Therefore, we describe the bit energy budget of “bit-0” in Equation (4).

(4) $$\begin{array}{ccc} E_{bit0} & = & P_{leak}\;\cdot \;\left(t_{low}+t_{bit}\right)+E_{spbit}\\ & = & \frac{V_{dd}^{2}}{R_{p}}\;\cdot \;\left(\frac{1}{2f_{SCL}}+t_{bit}\right)+\frac{1}{2}C_{b}V_{max}^{2} \end{array}$$

where the $P_{leak}$ is the power consumption caused by the leakage current. Accordingly, the bit energy budget of “bit-1” is formulated as Equation (5).

(5) $$\begin{array}{ccc} E_{bit1} & = & P_{leak}\;\cdot \;t_{low}+2E_{spbit}\\ & = & \frac{V_{dd}^{2}}{2R_{p}f_{SCL}}+C_{b}V_{max}^{2} \end{array}$$

Finally, we collect the bit energy budget to obtain the relationship between the bit rate and power consumption. The relationship is simply that the number of bits multiplied by the bit energy budget, as shown in Equation (6).

(6) $$\begin{array}{c} P_{diss}=B_{rate}\;\cdot \;\left[\alpha E_{bit0}+\left(1-\alpha \right)E_{bit1}\right] \end{array}$$

where we use the factor α∈[0,1] to represent the proportion of “bit-0” in all the transmitted data.

### 3.4. Optimal Resistance Problem

In this portion, we formulate the optimal resistance problem. Solving this problem can help us to find the optimal pull-up resistance in practical applications so as to make a trade-off between the power consumption and the data rate.

From the results in Figure 7, the power consumption and the data rate both decrease over the increasing $R_{p}$. The optimal resistance problem specifies finding an optimal $R_{p}$ value that obtains as much reduction as possible in the power consumption and as little sacrifice as possible to the data rate. We formulate the optimal resistance problem as follows.

(7) $$\mathrm{minimize}\;f_{o}\left(R_{p}\right)=\frac{P_{diss}\left(R_{p}\right)}{B_{rate}\left(R_{p}\right)}$$

subject to:

(8) $$\frac{t_{r}}{\ln\frac{7}{3}\;\cdot \;C_{b}}<R_{p}<\frac{3}{7}R_{load}$$

Before solving this problem, we should conduct practical measurement to get the function of $P_{diss}(R_{p}$) which depends on the fabrication of the practical PCB and the physical characteristics of the specific Sensor ICs on the PCB. The measured plot of $P_{diss}(R_{p}$) is shown in the evaluation section (Figure 11e). By solving this problem, the optimal $R_{p}$ can be selected to minimize the objective function $f_{o}\left(R_{p}\right)$. Since $f_{o}\left(R_{p}\right)$ is the rate of the power consumption to the bit rate, the optimal $R_{p}$ represents the minimum average bit energy budget.

## 4. Implementation and Evaluation

### 4.1. Implementation

Thanks to the potential waveshaping function in existing chips, we can easily implement *Turbo* by changing the two pull-up resistors on the bus. Although some I${}^{2}$C master chips have internal pull-up resistors, to implement *Turbo*, we disable the internal resistors and use external resistors. The value of pull-up resistors can be determined by the solution of the optimal resistance problem. Next, the implementation of the signal model and the optimal resistance problem can be simply realized by Matlab codes. We utilize the fminbnd function [19] in Matlab Optimization Toolbox to solve the problem. The simple implementation benefits that *Turbo* can be rapidly implemented on existing devices, even if the PCBs have been made. Moreover, the firmware in the devices does not need any modification as *Turbo* is harmless to the I${}^{2}$C standard.

### 4.2. Evaluation Overview

Our evaluation includes two portions. First, we evaluate the quality of I${}^{2}$C communication with the implementation of *Turbo*. Further, we solve the optimal resistance problem and see how the optimal value is affected by various parameters.

**Figure 10:**
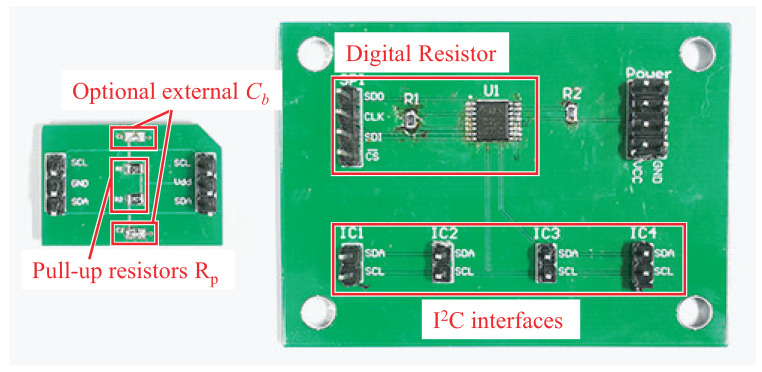
Experiment platforms. The left is a mini-test board, while the right is a multi-chips test board.

We build two platforms for I${}^{2}$C experiments as shown in Figure 10. First, the mini-test board can be use for simple two-chips I${}^{2}$C experiments that provide the proof-of-concept of *Turbo*. Second, the multi-chips test board faces to the comprehensive evaluations on multi-chips cases. We can solder desired pull-up resistors and optional external bus capacitors to imitate communication circumstances in different devices. Note that the bus capacitance $C_{b}$ is difficult to be decreased since it is determined by the nature of the bus lines. However, we can add $C_{b}$ by connecting an external capacitor to the bus. In the multi-chips test board, we employ the digital resistor circuit that enables us adjusting the pull-up resistors digitally by the control of an processor. The digital resistor is mainly composed by the potentiometer, AD5262 [20]. In the evaluation, the I${}^{2}$C buses on the two platforms both work at $V_{dd}$ = 3.3 V and $f_{SCL}$ = 100 KHz.

### 4.3. Quality of Communication

We conduct experiments to evaluate the *Turbo* performance, in terms of bit error rate (BER), bit energy budgets ($E_{bit0}$ and $E_{bit1}$), bit rate ($B_{rate}$) and bus power dissipation $P_{diss}$. We consider four off-the-shelf ICs in two types, i.e., with or without Schmitt triggers inside the chip. For each IC, we evaluate the metrics at a range of the pull-up resistance $R_{p}$. The parameters of each IC are shown in Table 1. The maximum standard resistance $R_{p(max\_std)}$ is calculated according to the standard via the factor $C_{b}$ that can be obtained with an oscilloscope to observe the signal rising time. Considering *Turbo* in various devices that have different $C_{b}$, we add the external bus capacitance 50 pF, 100 pF and 150 pF to the tests for 24AA08, Si7013 and PCF8574, respectively.

#### 4.3.1. Bit Error Rate

**Figure 11:**
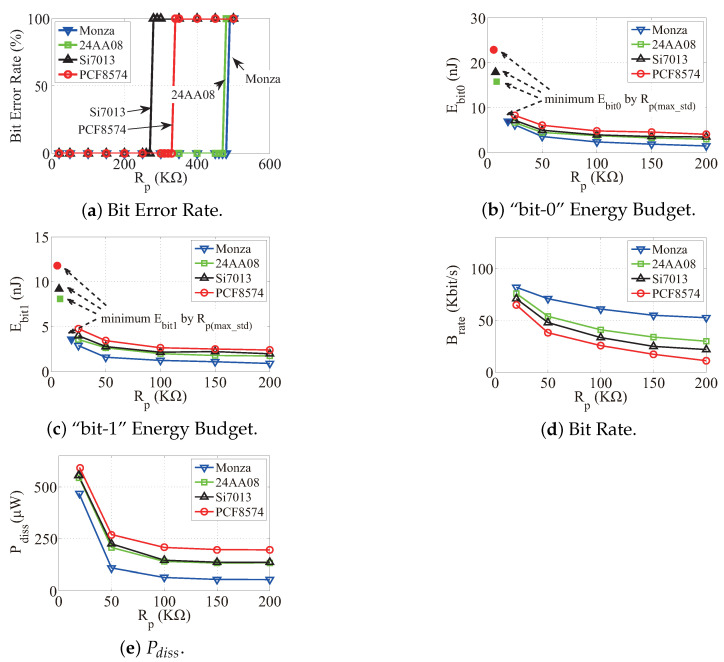
Experiment results of *Turbo*. The BER over different $R_{p}$ is shown in (**a**). We use “100% BER” to illustrate the case in which there are no bits (no matter the bits are correct or not) that can be transmitted via bus lines since both the SDA and SCL lines fail to work. The bit energy budget over different $R_{p}$ is shown in (**b**,**c**). We also mark the bit energy budget associated with the maximum standard resistance $R_{p(max\_std)}$ as a comparison in (**b**,**c**). As the sink rate of energy budgets becomes slow along with $R_{p}$, we recommend an empirical value $R_{p}$ = 50 KΩ as a general resistance selection for low power purpose. The maximum bit rates achieved with different $R_{p}$ values are shown in (**d**), and the overall power dissipation of I${}^{2}$C bus over different $R_{p}$ is shown in (**e**).

In the experiment, each IC is the slave role in the communication and is controlled by the master processor MSP430F2132 [18] working at 3.3 V supply voltage. The processor sends predefined data to each IC over hundred thousand times. The IC then executes established certain operations according to the data. For example, Monza will respond a fixed register value to the processor. Thus, the processor can evaluate the BER in communication. We repeat this process over different $R_{p}$. The results are shown in Figure 11a.

We observe that the BER of each IC polarizes (i.e., 0% or 100%). Specifically, the BER is zero when $R_{p}$ is less than a threshold, while the BER jumps to 100% (i.e., the case where no data bits are received) once $R_{p}$ is above the threshold. As we explained, the threshold is determined by the inside waveshaping module. When $R_{p}$ increases, the maximum peak voltage decreases. If the voltage is below the threshold, the waveshaping module cannot recover the signal and consequently causes communication failures. The thresholds are different among the ICs. For the ICs with Schmitt triggers (e.g., Monza and 24AA08), the thresholds are close since the triggers are compliant to the standard including the voltage definitions. For the ICs without the trigger, the register is optimized to keep data, not for the waveshaping. Thus, the voltage thresholds may not be same with the standard definitions.

#### 4.3.2. Bit Energy Budget

We evaluate $E_{bit0}$ and $E_{bit1}$ by experiments. We measure $t_{bit}$ and $V_{max}$ by an oscilloscope and calculate the energy budget according to Equations (4) and (5), respectively. We repeat the experiment at a range of $R_{p}$ values. The results are shown in Figure 11b,c, respectively. From the results, we observe that $E_{bit0}$ and $E_{bit1}$ both decrease along with the increase of $R_{p}$ because the leakage current is reduced. Further, larger $C_{b}$ incurs higher $E_{bit0}$ and $E_{bit1}$ when $R_{p}$ keeps constant. The reason stems from the fact that larger $C_{b}$ implies more switching dissipation. Furthermore, $E_{bit0}$ is higher than $E_{bit1}$ in the same condition of $R_{p}$ and $C_{b}$. This is because the leakage current is typically higher than the switching dissipation.

#### 4.3.3. Bit Rate

This evaluation considers the bit rate $B_{rate}$ associated with varying $R_{p}$. The experiment is conducted by measuring $t_{bit}$ by an oscilloscope. Then, the bit rate can be calculated according to Equation (3). We show the results in Figure 11d. From the results, we observe that the bit rate has a negative relationship with both $R_{p}$ and $C_{b}$. This is because the equivalent RC circuit on the bus will delay the propagation of signals. Specifically, outputting a high level voltage can be treated as a process to charge the bus capacitor.

### 4.4. Optimal Resistance with Various Parameters

**Figure 12:**
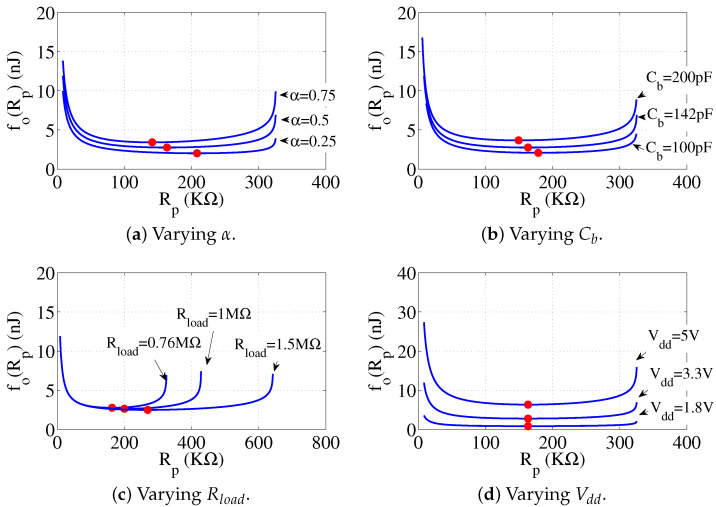
The simulation results of optimal resistance values over different parameters. Each optimal value is marked by a red point. $f_{o}\left(R_{p}\right)$ represents the average bit energy budget. In (**ab**), the optimal $R_{p}$ decreases along with the increase of α and $C_{b}$, respectively. In (**c**), $R_{load}$ does not change the curve on the ordinate and just prolong the curve on the abscissa. In (**d**), higher $V_{dd}$ will not impact the optimal $R_{p}$ value and only introduces higher power consumption. As $f_{o}\left(R_{p}\right)$ is almost same when $R_{p}$ = 100 KΩ to 250 KΩ in (**a**–**d**), we recommend an empirical value $R_{p}$ = 100 KΩ as a general resistance selection.

We can select the optimal $R_{p}$ value by solving the optimal resistance problem in order to obtain the minimum bit energy budget. In this portion, we evaluate the optimal value over varying α (the proportion of “bit-0” in the test data), $C_{b}$, $R_{load}$ and $V_{dd}$, respectively. The evaluation is conducted by simulations on the IC 24AA08 and the basic parameters are shown in Table 2. For each simulation, we only tune a single parameter and keep the others same. Figure 12 shows the simulation results with various parameters.

From the results, we also observe that the value of $f_{o}\left(R_{p}\right)$ keeps almost same in a range of $R_{p}$, roughly 100 KΩ to 250 KΩ. Hence, we recommend an empirical value $R_{p}$ = 100 KΩ since the bit energy budget is near to the minimum, while the data rate could be much higher that the minimum case. Taking 24AA08 as an example, the average bit energy budget is reduced to 24.3% compared to the maximum standard resistance $R_{p(max\_std)}$, meanwhile only sacrificing 54.8% of the bit rate. As a comparison, the bit energy budget and the reduced bit rate are 23% and 63.3%, respectively, when the optimal $R_{p}$ in Table 1 is selected. The recommended value would benefit engineers selecting a general pull-up resistor value in practical applications.

## 5. Discussion

**Different I${}^{2}$C Speed Modes.** The I${}^{2}$C standard supports several speed modes, including the standard mode (up to 100 Kbit/s), the fast mode (up to 400 Kbit/s) and the high-speed mode (up to 3.4 Mbit/s). We mainly discuss and evaluate *Turbo* in the standard mode. We believe that *Turbo* adapts to other modes because the standard specifies that the ICs of higher modes should incorporate the Schmitt triggers at their inputs and thus automatically gain the waveshaping ability.

**Reduction of Clock Frequency.** In digital circuits, reduction of clock frequency is a basic way to lower power consumption. However, this solution cannot work well because the leakage current is the major power consumption in I${}^{2}$C. Although the clock reduction introduces lower switching dissipation (dynamic power), the reduced clock frequency incurs more leakage consumption due to longer time to keep the bus at logic low that causes static leakage current. In future work, we will attempt to combine the clock reduction and the resistance increment to investigate a way to further reduce the power consumption in I${}^{2}$C communication.

**Compatibility to Existing Chips.***Turbo* obeys the I${}^{2}$C standard definitions that the high voltage $V_{high}$ refers to above 70% of $V_{dd}$ and the low voltage $V_{low}$ is below 30% of $V_{dd}$. In practice, however, we observe that some existing chips do not exactly follow the definitions. For example, the chip MSP430FR5969 [23] typically has $V_{high}$ = 65%$\;\cdot \;V_{dd}$ and $V_{low}$ = 35%$\;\cdot \;V_{dd}$. This is because each chip meets special considerations in the design. Specifically, I${}^{2}$C and another serial bus share a same I/O port on MSP430FR5969, in which the designers have to consider compatibility of the voltage definitions to both buses. The fact may introduce a negative impact that different chips may have different effects when *Turbo* is implemented. We believe that the effect is slight as the voltage definitions in existing chips would not be hugely different away from the I${}^{2}$C standard.

**Dynamic Data Rate Demand.** The device may require the I${}^{2}$C bus performing high bit rate transmissions in some cases and dynamically adopting low power I${}^{2}$C when the system available energy is weak. However, the maximum bit rate is affected by the value of $R_{p}$ that is implemented by a resistor in hardware. In order to meet this case, the resistance should be dynamically controlled by software in the processor. To achieve this, the pull-up resistors should be digital resistors or digital potentiometers as shown in Figure 10.

## 6. Related Work

**Passive Sensing Devices.** This paper involves sensor devices. The most of research efforts on this topic concentrate on radio to mitigate the energy problem. Traditionally, the radio drains major power consumption (several mWs) in sensing devices, which is much higher than the power consumption regarding bus communication to operate sensors. In the past decade, backscatter communication dramatically reduces the power consumption of the radio down to μWs [7,8,24], and a series of efforts are proposed to optimize energy utilization efficiency [25,26,27,28], the energy cost of energy-level detection hardware [29], and even eliminate the power-consuming processor from sensing devices [1,2,3]. Thus, it is possible to make the devices passive with micro-energy harvesting. However, the inefficiency of energy harvesting causes that the energy is still scarce in passive devices [30,31]. However, for a long time, the weight of bus communication power consumption is overlooked. This paper at the first time points out this problem and takes steps to reduce the energy overhead in bus communication to access multiple sensors.

**Multi-sensors Connections.** In this paper, we advocate the I${}^{2}$C bus to connect multiple sensors. As we know, other general purpose digital buses like SPI can be also used to connect such sensors. However, the I${}^{2}$C bus has its unique advantage in terms of the simple topology on hardware connection, which towers over other buses in multi-sensors case. For example, the SPI bus requires at least 3 + *n* lines (*n* is the number of sensors), which occupies additional processor ports that is a constrained resource in passive sensing devices. In addition, I${}^{3}$C [32] has been released as an evolution featuring lower power consumption than I${}^{2}$C. To implement I${}^{3}$C, the master processors have to support the specific functions. The existing processors employed in current passive sensing devices, however, do not support I${}^{3}$C. This means that I${}^{3}$C will not be implementable immediately to the existing devices. Therefore, we choose I${}^{2}$C and we believe that the proposed *Turbo* facilitates existing passive sensing devices with the simple topology and energy optimization simultaneously.

**Power Optimization for I${}^{2}$C.** Currently, the effective methods to optimize power consumption of I${}^{2}$C stem from the fact that the energy budget to transmit “bit-0” is higher than the energy for “bit-1” as the leakage current consumption is more obvious in “bit-0” transmission.

*Disabling Pull-Up Resistors.* The forum thread [33] discusses a question what happens if the pull-up resistors are removed. The omitted resistors are equivalent to $R_{p}$ = ∞Ω that incurs zero leakage current. While the I${}^{2}$C standard partly allows the omission, it introduces two main problems. First, the approach is applied to a special case that in ultra fast mode, the data is transmitted in only one direction between two chips. Second, the approach is available only for a part of I${}^{2}$C-interfaced chips that the I/O circuit supports the push–pull output architecture. In this paper, the proposed *Turbo* considers the general cases that allow bidirectional communication among multi-chips. Further, the authors in [34] propose an approach using a software I${}^{2}$C implementation which disables pull-ups when the master sends “bit-0”. However, software I${}^{2}$C requires the processor staying active to simulate the protocol, which often incurs higher power consumption. Although this overhead can be mitigated by hardware implementation, it needs to modify the master and results in being not implementable to the existing devices.

*Coding Techniques.* Another attempt is to encode data transmissions with more “bit-1” than “bit-0”. The coding techniques can be classified into two categories. (1) The software coding [35] which is implemented by software in the ICs. This technique often is employed in data storage that the data can be freely encoded, but is restricted in many scenarios. For example, commands and sensing results in most of the sensors are not admitted to be encoded. In addition, the I${}^{2}$C clock on the SCL line cannot be encoded. (2) *The hardware coding* [36] leverages an encoder/decoder pair which is placed at the transmitter and receiver side, respectively. This technique has the merit of user transparency since the encoded data will be decoded before received, but mostly causes more energy consumption in I${}^{2}$C communication. This is because the original data has to be sent twice from the transmitter to the encoder and from the decoder to the receiver. Differing from *Turbo*, this technique is often used as relays for long distance communication but not for reducing the consumption, and it has higher hardware cost and poorer compatibility than *Turbo*.

*Varying Pull-Up Resistors.* The technical article [37] shows the signal waveforms associated with a range of $R_{p}$ values by conducting several experiments. Further, the article analyzes the signal patterns over the increase of $R_{p}$. Compared with the experiments in this paper, there are three differences. First, the $R_{p}$ range in the article is within the range specified by the standard. Second, the article does not discuss neither the over-sized $R_{p}$ values nor the reasons of waveform effects. Third, the article only discusses the data rate over $R_{p}$, but does not take the power consumption into account. In this paper, we concern the I${}^{2}$C power consumption in low power devices. We investigate the waveform effects from not only the standard $R_{p}$ range, but also the over-sized $R_{p}$ values. Further, we interpret the essential reasons of the effects by our technical observations.

## 7. Conclusions

In this paper, we propose *Turbo* to reduce power consumption of I${}^{2}$C communication in order to achieve low power multi-sensors accesses in passive sensing devices while keeping the simple connections. The basic idea is leverage a waveshaping module for signal recovery. Then, we observe the potential waveshaping function in off-the-shelf sensor chips so that *Turbo* can be implemented directly by only changing the value of pull-up resistors. Further, we determine the effective resistance selection range and show that *Turbo* achieves robust communication within the resistance selection range. We also propose the physical signal model for I${}^{2}$C bus to describe the relationship between the energy consumption and the data rate in *Turbo*. Finally, we formulate the optimal resistance problem as an optimization problem to seek the best pull-up resistor value. Our evaluation shows that when the optimal resistance is selected, the energy consumption is minimized to 23.1% of the standard value. We also recommend an empirical resistance 50 KΩ that achieves near minimum energy consumption in practical cases.

The future work of *Turbo* includes two directions. First, we will optimize the design of *Turbo*. For example, a better design could be IC independent that the waveshaping module should be outside the ICs. Second, we will further investigate a way to improve the accuracy of the signal model.

## Figures and Tables

**Table 1:** Experiment parameters in the evaluation and the comparison between the standard and *Turbo*. The symbol “•” represents the IC with the Schmitt trigger, while the symbol “∘” indicates the IC without the Schmitt trigger.

Test IC		• Monza [16]	• 24AA08 [17]	∘ PCF8574 [21]	∘ Si7013 [22]
$C_{b}$		63pF	142pF	206pF	161pF
**Standard**	$R_{p(\max\_\mathrm{std})}$	18.7 KΩ	8.3 KΩ	5.7 KΩ	7.3 KΩ
	$E_{\mathrm{bit}0}$	7 nJ	15.8 nJ	22.9 nJ	17.9 nJ
	$E_{\mathrm{bit}1}$	3.6 nJ	8.1 nJ	11.8 nJ	9.2 nJ
	$B_{\mathrm{rate}}$	90.9 Kbit/s	90.9 Kbit/s	90.9 Kbit/s	90.9 Kbit/s
**Turbo**	optimal $R_{p}$	200 KΩ	163.4 KΩ	148 KΩ	158.1 KΩ
	$E_{\mathrm{bit}0}$	2.2 nJ	4.1 nJ	5.6 nJ	4.6 nJ
	$E_{\mathrm{bit}1}$	0.7 nJ	1.4 nJ	1.9 nJ	1.5 nJ
	$B_{\mathrm{rate}}$	52.7 Kbit/s	33.4 Kbit/s	17.6 Kbit/s	25.1 Kbit/s

**Table 2:** Simulation parameters.

$V_{\mathrm{dd}}$	$f_{\mathrm{SCL}}$	$C_{b}$	$R_{\mathrm{load}}$	α	Δt
3.3 V	100 KHz	142 pF	0.76 MΩ	0.5	0

## Data Availability

The data presented in this study are available on request from the first author.

## Abbreviations

The following abbreviations are used in this manuscript:

IoT	Internet of Things
COTS	Commercial Off-the-shelf
BER	Bit Error Rate
SPDT	Single-Pole Double-Throw
SPST	Single-Pole Single-Throw
TX	Transmit
RX	Receive
IC	Integrated Circuit
LED	Light-Emitting Diode
